# Expression of DDX3 Is Directly Modulated by Hypoxia Inducible Factor-1 Alpha in Breast Epithelial Cells

**DOI:** 10.1371/journal.pone.0017563

**Published:** 2011-03-23

**Authors:** Mahendran Botlagunta, Balaji Krishnamachary, Farhad Vesuna, Paul T. Winnard, Guus M. Bol, Arvind H. Patel, Venu Raman

**Affiliations:** 1 Department of Radiology and Radiological Sciences, Johns Hopkins University School of Medicine, Baltimore, Maryland, United States of America; 2 Medical Research Council Virology Unit, University of Glasgow, Glasgow, United Kingdom; 3 Department of Oncology, Johns Hopkins University School of Medicine, Baltimore, Maryland, United States of America; University of Nebraska Medical Center, United States of America

## Abstract

DEAD box protein, DDX3, is aberrantly expressed in breast cancer cells ranging from weakly invasive to aggressive phenotypes and functions as an important regulator of cancer cell growth and survival. Here, we demonstrate that hypoxia inducible factor-1α is a transcriptional activator of *DDX3* in breast cancer cells. Within the promoter region of the human *DDX3* gene, we identified three putative hypoxia inducible factor-1 responsive elements. By luciferase reporter assays in combination with mutated hypoxia inducible factor-1 responsive elements, we determined that the hypoxia inducible factor-1 responsive element at position -153 relative to the translation start site is essential for transcriptional activation of DDX3 under hypoxic conditions. We also demonstrated that hypoxia inducible factor-1 binds to the DDX3 promoter and that the binding is specific, as revealed by siRNA against hypoxia inducible factor-1 and chromatin immunoprecipitation assays. Thus, the activation of *DDX3* expression during hypoxia is due to the direct binding of hypoxia inducible factor-1 to hypoxia responsive elements in the *DDX3* promoter. In addition, we observed a significant overlap in the protein expression pattern of hypoxia inducible factor-1α and DDX3 in MDA-MB-231 xenograft tumors. Taken together, our results demonstrate, for the first time, the role of *DDX3* as a hypoxia-inducible gene that exhibits enhanced expression through the interaction of hypoxia inducible factor-1 with hypoxia inducible factor-1 responsive elements in its promoter region.

## Introduction

Human *DDX3* is a member of the DEAD-box family of RNA helicases and is located on the X chromosome [1]. DEAD-box RNA helicases have been shown to function in RNA metabolism including translation, ribosome biogenesis, pre-mRNA splicing, and nucleo-cytoplasmic RNA transport [2]–[4]. Human DDX3 shares significant amino-acid sequence homology with orthologs from several species including yeast (Ded1), Drosophila (Bel), Xenopus (An3), and murine (PL10) [5]–[8]. Thus, natural selection of an ancestral DDX3 protein with characteristics that have been passed along to higher organisms is an indication that this protein is involved in cellular pathways that are essential to survival. In humans DDX3 has a function in folliculogenesis as its deletion or dysfunction represents an important genetic cause of primary amenorrhea or impairment of female fertility [9]. Recently DDX3 has been the focus of a great deal of research because of its involvement in the replication of the human immunodeficiency virus, hepatitis C virus, and poxviruses [10]–[16].

Recent work indicates that DDX3 can participate in the transcriptional regulation of a diverse set of genes involved in apoptosis and cellular transformation in ways that impact cancer progression [17]. It has been demonstrated that apoptosis was triggered by DDX3 modulated transactivation of the expression of *p21^waf1/cip1^* gene [18]. The modulation of *p21^waf1/cip1^* gene expression accounts for the growth-suppressive effect of DDX3 in hepatocellular cell lines. On the other hand, our work has shown that over-expression of DDX3 brought about a cellular transformation leading to the down-regulation of E-cadherin expression in immortalized breast epithelial cells (MCF 10A cells) [19]. Down-regulation of E-cadherin is a marker of an epithelial mesenchymal transition (EMT) phenotype, which is associated with cancer progression in several cancers [19]–[22]. We also found that DDX3 expression is directly correlated with tumorigenesis in a panel of breast epithelial cell lines ranging from non-tumorigenic (low DDX3) to highly aggressive cancer phenotypes (high DDX3) [19]. In MDA-MB-231, a highly aggressive metastatic breast cancer cell line, DDX3 was found within an anti-apoptotic complex consisting of glycogen synthase kinase 3 (GSK3) and cellular inhibitor of apoptosis 1 (c-IAP1), which is an indication of its importance in the therapeutic resistance of tumor cells to TRAIL receptor antibody therapy [23]. Thus, DDX3 has diverse functions in a variety of cell types, in breast cancer cells DDX3 augments cell proliferation whereas in hepatocellular carcinoma cells it promotes growth arrest and tumor suppressing activities.

Hypoxia is a major characteristic of solid tumors and a condition that affects genome-wide changes in gene expression, which greatly impacts cellular and tumor tissue physiology particularly respiration and metabolism [23]–[29]. Expression of hypoxia-responsive genes is predominately regulated by hypoxia inducible factors (HIFs) [23], [25]–[34]. HIFs are basic helix-loop-helix/PAS transcription factors consisting of an alpha subunit (e.g., HIF-1α) and a β subunit, i.e., aryl hydrocarbon receptor nuclear transporter (ARNT) [30]. HIF-1 is expressed in most tissues and functions as the principal transcriptional regulator of most HIF responsive element (HRE) containing genes while HIF-2 exhibits restricted expression and a more limited scope of regulation [35]. Under normoxic conditions, HIF-1α and 2α are subjected to ubiquitination and proteasomal degradation [36]. However, under hypoxic conditions, HIF-1α or 2α heterodimerize with ARNT forming HIF-1 and HIF-2, which bind to hypoxic response elements (HREs) within the promoters of target genes to promote transcription [37], [38]. HREs have been found in a remarkably large set of genes that affect many different genetic programs, including proliferation, differentiation, tissue-specific responses, and cell death [39]. During our studies of *DDX3* we discovered putative HRE sequences within its promoter. Therefore, we hypothesized that the hypoxic microenvironment within solid tumors activates the expression of *DDX3*.

In this study, we investigated whether expression of *DDX3* gene is up-regulated by HIF-1α or HIF-2α in response to hypoxia in human breast epithelial cell lines. Our data provide evidence that hypoxic induction of the *DDX3* gene is mediated by transactivation of *DDX3* promoter by HIF-1α through a consensus HRE binding site.

## Results

### Hypoxia regulates DDX3 expression in breast cancer cells

**Figure 1:**
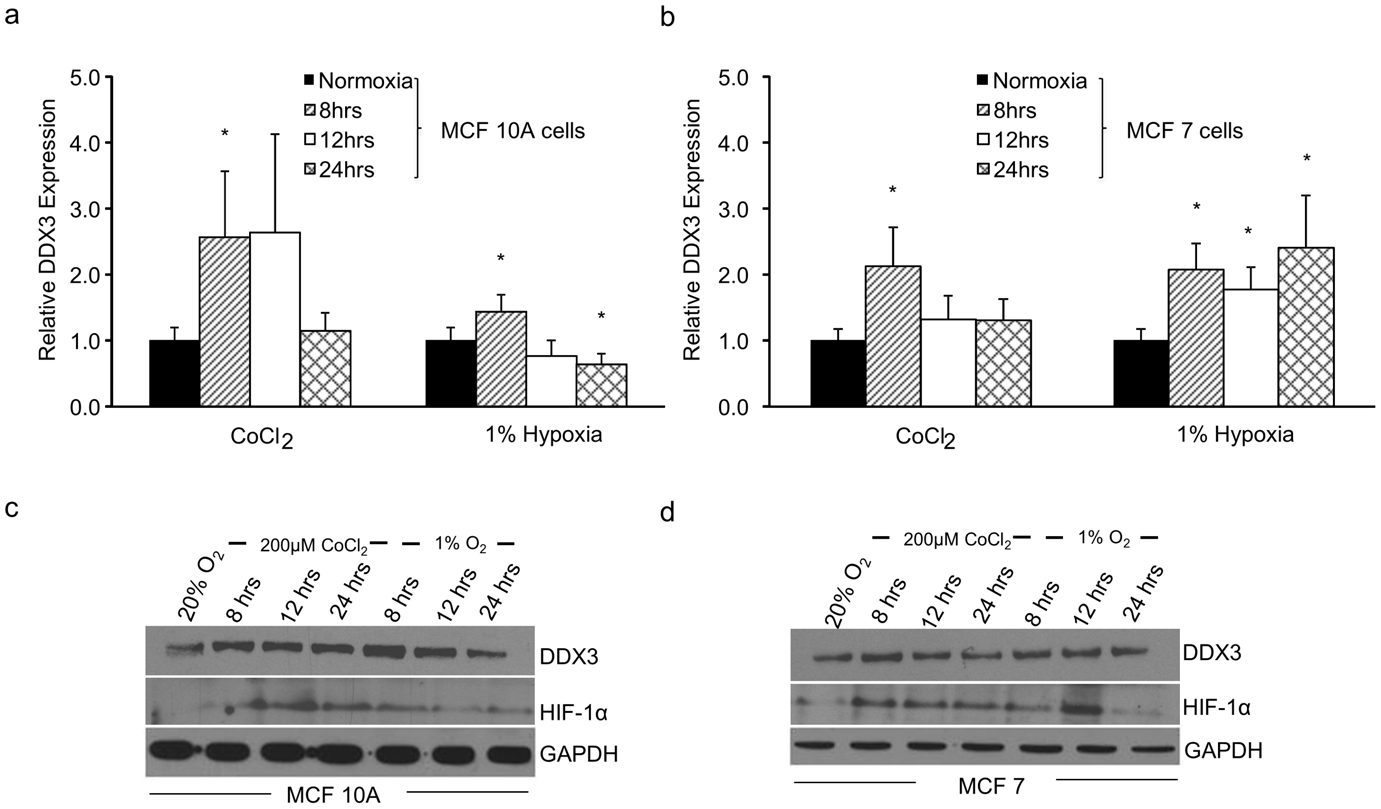
Effect of hypoxic conditions on the expression of the DDX3 gene in breast epithelial cells. MCF 10A (a & c) and MCF 7 (b & d) cells were cultured under normoxic conditions (20% O_2_) or subjected to 200 µM CoCl_2_ or 1% O_2_ for 8, 12 and 24 h (a & b) qRT-PCR analysis was performed using specific primers for human DDX3 and HPRT as an internal normalization control. The expression level under normoxia was set to 1. DDX3 protein levels (c & d) in cell lysates of MCF 10A and MCF 7 cells were determined by immunoblots using anti-DDX3 antibodies. In these cases, HIF-1α and GAPDH served as controls indicating hypoxic conditions and equal protein loading respectively. Error bars represent ±SD.

To investigate whether the expression of *DDX3* is regulated by hypoxia, we compared the mRNA levels of DDX3 in cells cultured under normoxic (20% O_2_) and hypoxic (1% O_2_) or hypoxia-mimetic (cobalt chloride: CoCl_2_) conditions. A time course (8, 12, and 24 h time points) experiment in MCF 10A cells demonstrated that the induction of DDX3 mRNA was transient (Figure 1a). DDX3 mRNA expression levels increased to 2.3 and 1.4 fold in MCF 10A cells at 8 h following exposure to CoCl_2_ or 1% O_2_ respectively but after 12 h in 1% O_2_ and at 24 h in CoCl_2_ DDX3 mRNA levels returned to or fell below normoxic levels (Figure 1a). To confirm the generality of this effect, we exposed weakly tumorigenic MCF 7 cells, to CoCl_2_ or 1% O_2_ and measured DDX3 mRNA levels. In the case of 1% O_2_ treatment, DDX3 mRNA levels were up-regulated approximately two fold relative to that of normoxic levels, at all time points tested (Figure 1b). In contrast, following CoCl_2_ exposure, levels of DDX3 mRNA reached a similar 2.0 fold increase at 8 h and then decreased to approximately normoxic levels at 12 and 24 h (Figure 1b). We next proceeded to test this result at the protein level. MCF 10A and MCF 7 cells were treated with either CoCl_2_ or 1% O_2_ or left under normoxia and total protein extracts were prepared and analyzed for DDX3 and HIF-1α protein levels by immunoblot. The time course examined was identical as that used during the mRNA experiments. In MCF 10A cells, relative to normoxic conditions, DDX3 protein levels increased by 8 h of CoCl_2_ exposure and remained elevated throughout the duration of the experiment. However, with 1% O_2_ exposure DDX3 protein levels in MCF 10A cells increased at 8 h and then began to decrease by 12 h reaching approximately normoxic conditions by 24 h (Figure 1c). Relative to normoxic levels, DDX3 protein levels in MCF 7 cells increased by 8 h of CoCl_2_ or 1% O_2_ treatment, stayed elevated at 12 h during 1% O_2_ treatment but started to decline at this time point during CoCl_2_ treatment, and then declined back to normoxic levels by 24 h of CoCl_2_ treatment and to levels approaching normoxic levels during 1% O_2_ treatment (Figure 1d). HIF-1α proteins levels were used as a positive control indicative of hypoxic conditions. Overall, these data demonstrate that hypoxia induces DDX3 expression of both mRNA and protein levels in normal and malignant breast cells.

### DDX3 gene expression is dependent on HIF-1α stabilization during CoCl_2_ treatment

**Figure 2:**
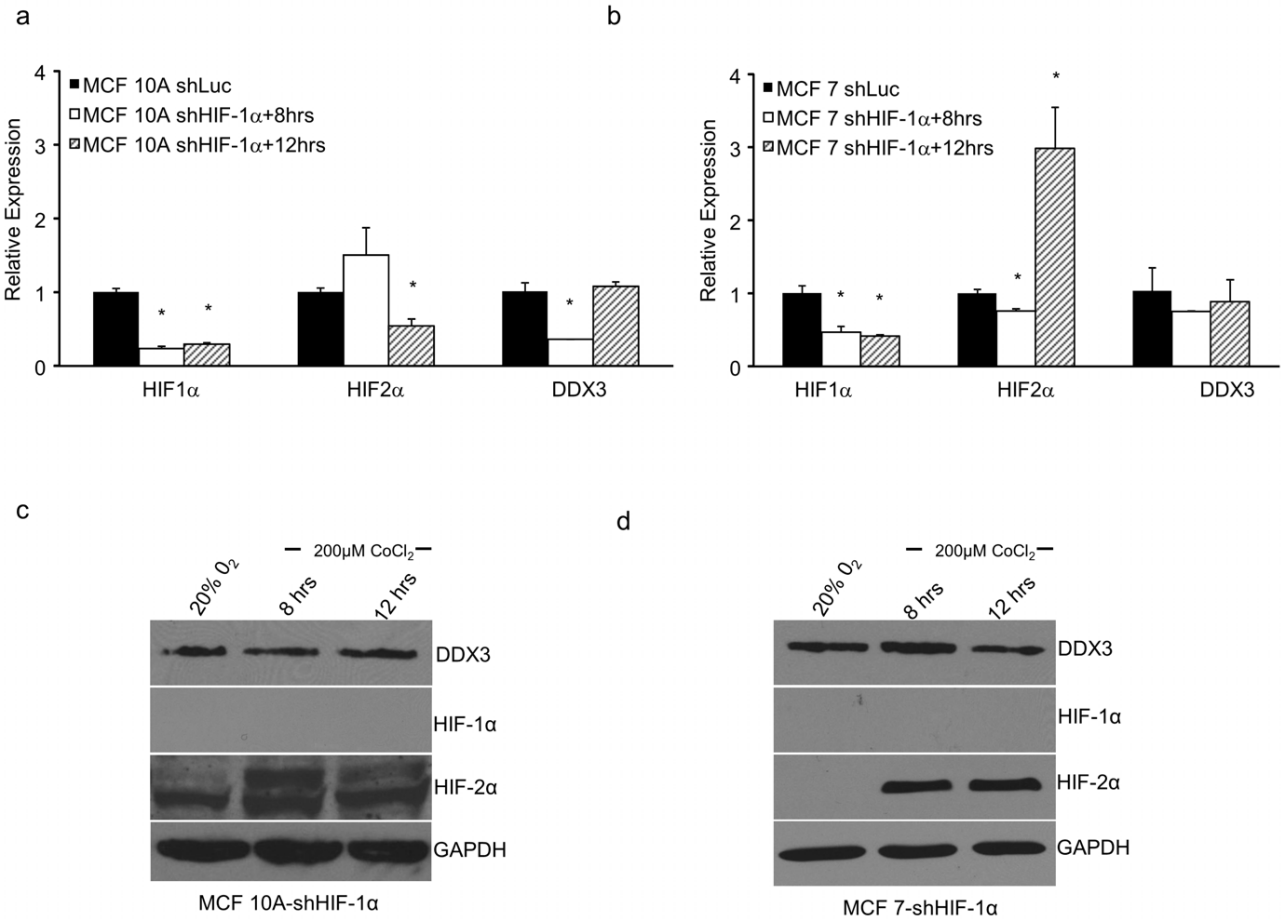
Specificity of HIF-1α dependent activation of the DDX3 gene. Stable MCF 10A and MCF 7-HIF-1α shRNA clones were generated. (a) MCF 10A-shHIF-1α and (b) MCF 7-shHIF-1α cells were subjected to 200 µM CoCl_2_ for the indicated times. qRT-PCR analysis was performed using specific primers for human DDX3, HIF-1α and HIF-2α. DDX3, HIF-1α, and HIF-2α protein levels in cell lysates of MCF 10A-shHIF-1α (c) and MCF 7-shHIF-1α (d) cells were examined by immunoblots using protein specific antibodies. GAPDH served as a loading control. Error bars represent ±SD.

To determine the role of HIF-1 or HIF-2 in regulating the expression of DDX3, HIF-1α expression was knocked down in MCF 10A and MCF 7 cells with shRNAs targeting HIF-1α, which generated MCF 10A-shHIF-1α and MCF 7-shHIF-1α cells. These stable cell lines were subjected to CoCl_2_ treatment and the mRNA levels of DDX3, HIF-1α, and -2α were determined 8 and 12 h later. As shown in Figure 2a and b, the presence of CoCl_2_ did not induce HIF-1α mRNA in MCF 10A-shHIF-1α or MCF 7-shHIF-1α cells, as compared to control cells. In contrast, 8 h of CoCl_2_ treatment caused an increase in HIF-2α mRNA in MCF 10A-shHIF-1α cells and an increase in HIF-2α at 12 h in MCF 7-shHIF-1α cells, indicating that the shRNA effect was specific to HIF-1α. Consistent with the repression of HIF-1α expression, DDX3 mRNA was not increased in either shHIF-1α expressing cell line under CoCl_2_ conditions. However, the level of DDX3 mRNA is slightly decreased at 12 h in MCF 10A and MCF 7 cell lines. We next performed immunoblot analysis on identical samples to determine whether the difference in the expression of HIF-1α, HIF-2α and DDX3 mRNA in these cell lines influences their protein expression. Figure 2c and d demonstrates that DDX3 protein levels mirrored the mRNA levels seen during hypoxic simulation in HIF-1α knockdown MCF 10A and MCF 7 cell lines while HIF-2α levels were up-regulated. These data provide the first direct demonstration of a specific regulation of an important RNA helicase gene by HIF-1.

### Characterization of human DDX3 promoter in MCF 7 cell line

**Figure 3:**
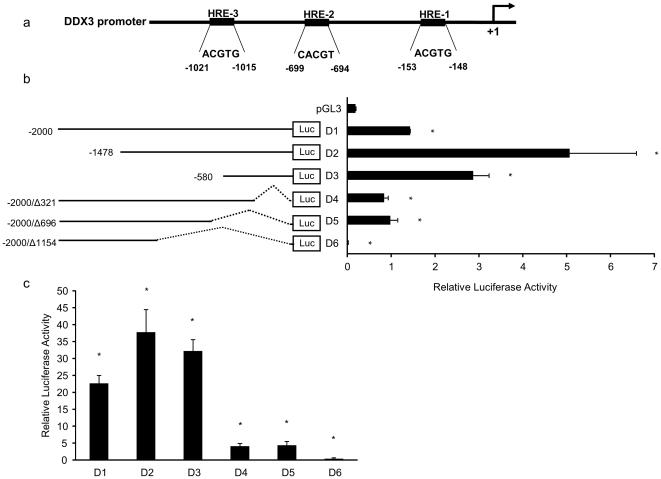
Characterization of the putative HRE containing sequences of the human DDX3 promoter. (a) Schematic representation of putative core HRE sequences in the promoter region of the DDX3 gene. (b) Linear representations of the DDX3 promoter-reporter constructs and the relative firefly luciferase activity of each in transient transfection assays in MCF 7 cells with pRRL-CMV (renilla luciferase expressing vector) as an internal control. After transfection cells were incubated in normoxia for 24 h Relative firefly luciferase activities are presented as a histogram at the right. (c) Effects of over-expression of HIF-1α in MCF 7 cells under normoxia. Cells were co-transfected with the DDX3 promoter-reporter constructs shown in (b), with pCDNA-HIF-1α (constitutive HIF-1α expressing vector), and pRRL-CMV as the internal control. Relative firefly luciferase activities are presented. Mean values from three independent transfections are shown. Error bars represent ±SD.

We next investigated whether regulation of *DDX3* by hypoxia occurs at the transcriptional level. To identify putative transcription factors involved in the regulation of *DDX3* gene expression, we retrieved the DDX3 promoter sequence (Acc. No NG_012830) and analyzed it at the Genomatix site (http://www.genomatix.com). At least three putative core HRE (A/GCGTG) sites were found within the 2.1 kb DDX3 promoter region at −153 (HRE-1), −699 (HRE-2) and −1021 (HRE-3), relative to the ATG translation start codon (Figure 3a). To assess the functional activity of DDX3 promoter, transfection experiments were performed in MCF 7 breast cancer cells, using different DDX3 promoter-reporter vector constructs. Figure 3b, left panel, depicts a schematic representation of the different promoter-reporter deletion constructs (D1 – D6) used. Our results indicate that there may be a repressor(s) within the −1478 to −2000 bp range (compare D1 expression to that of D2; Figure 3b). In addition, our D3 construct indicates that an enhancer(s) apparently is present within the −581 to −1478 region as reporter activity relative to that of the D2 construct dropped with the removal of this region. We also observed a large decrease in promoter activity with the deletion of proximal promoter regions as seen with a comparison of the reporter activity of constructs D4, D5 and D6 to that of D2 (Figure 3b). Thus, under the conditions used in MCF 7 cells, the 1.48 Kb promoter region was the most active of the reporter constructs. To test whether exogenous HIF-1α over expression can cause a stimulatory effect on DDX3 promoter activity in normoxic conditions, we co-transfected the DDX3 promoter-reporter vector constructs with a vector that provides for constitutive expression of HIF-1α. As shown in Figure 3c, under these conditions the pattern of reporter activities mirrored that seen in Figure 3B but the activities were increased by an order of magnitude. This data provides further direct evidence that HIF-1α is involved with the regulation of DDX3 expression.

### Identification of functional HREs in the DDX3 promoter

**Figure 4:**
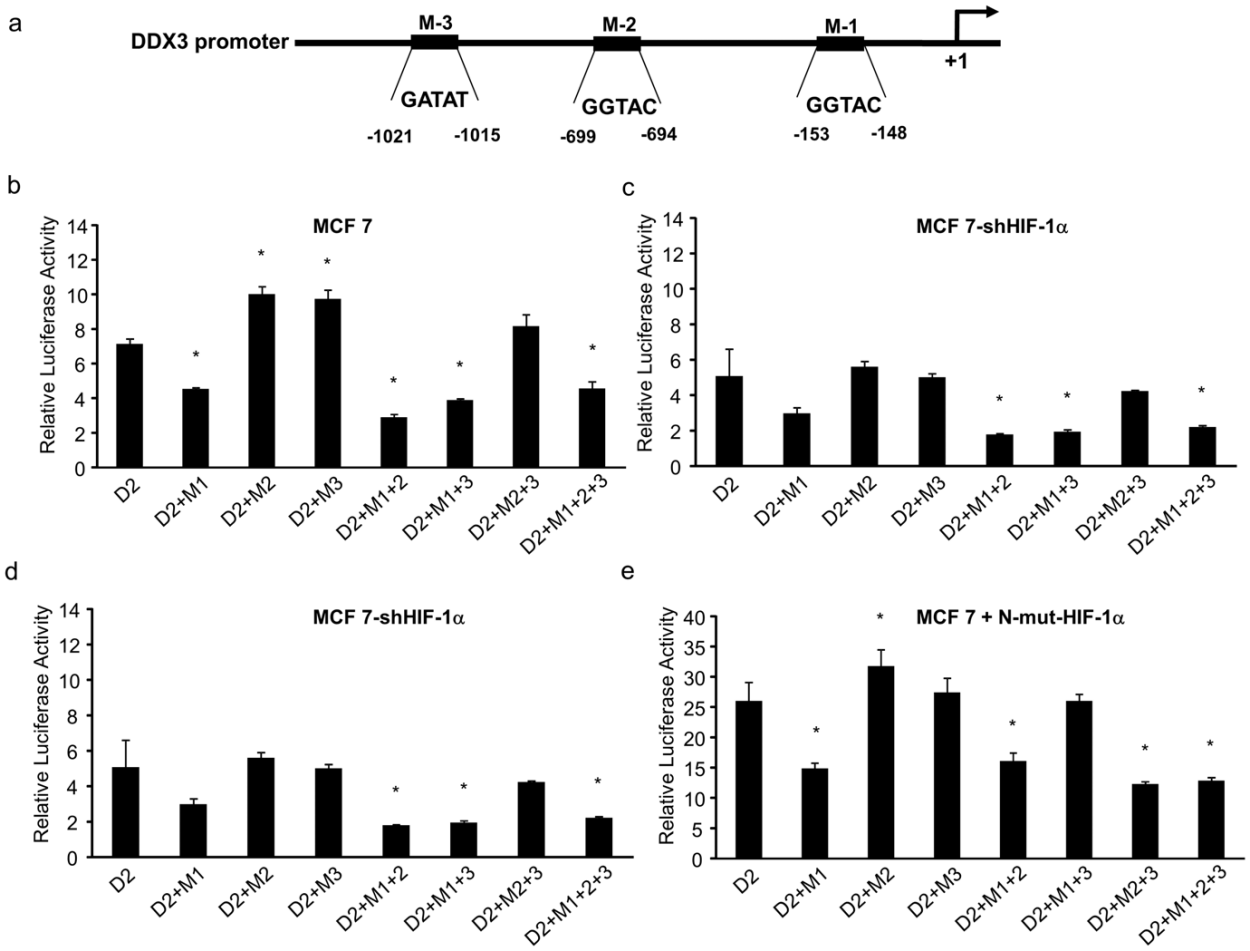
Identification of functional HRE in the DDX3 promoter. (a) The regions of the individually mutated HREs-1-3 (Mut1-3) are indicated schematically. MCF 7 and MCF 7-shHIF-1α cells were transfected with wild-type (D2), singly (M1, M2, M3), doubly (M1+M2, M1+M3, M2+M3) or triply (M1+M2+M3) mutated promoter-reporter constructs, as indicated. (b & c) After transfection MCF 7 or MCF 7-shHIF-1α cells were cultured under normoxia. (d) After transfection MCF 7 were cultured in presence of CoCl_2_. (e) Each transfection of MCF 7 cells included co-transfection with a constitutive HIF-1α expression vector. Histograms show relative firefly luciferase activity. Each experiment was performed in triplicate on at least three separate occasions. Error bars represent ±SD.

Site directed mutagenesis was performed within individual HREs (HRE-1: M1 or HRE-2: M2 or HRE-3: M3) as well as in combinations of HREs (M1+M2 or M1+M3 or M2+M3 or M1+M2+M3) to produce the 7 promoter-reporter constructs depicted in Figure 4a. To determine if HIF-1 binds to any or all of these putative HRE sequences, mutated promoter-reporter constructs were transiently transfected into MCF 7 cells and the cells were incubated for 24 h under normoxic conditions and then harvested for luciferase assay. Again, the activity of the D2 construct was relatively high in MCF 7 cells (Figure 4b). However, relative to the D2 construct, we found a 35% reduction in the mutated HRE-1 (M1) promoter activity (Figure 4b). In contrast, disruption of HRE-2 (M2) or HRE-3 (M3) enhanced luciferase activity under normoxic conditions. We further demonstrated that combinations of double or triple mutation constructs that included mutated HRE-1 (M1), i.e., M1+M2 or M1+M3 or M1+M2+M3, reduced reporter activity to levels similar to or lower than that of M1 activity (Figure 4b). In addition, exclusion of M1 as a double mutation, i.e., M2+M3, did not inhibit reporter activity (Figure 4b). To determine whether the basal reporter expression from DDX3 promoter-report constructs in normoxia is due to constitutive expression/stabilization of HIF-1α, we transiently transfected D2 and the mutated promoter-reporter constructs into MCF-7-shHIF-1α cells. As shown in Figure 4c, under shHIF-1α conditions the reporter activities shown in Figure 4b were decreased in all cases while the pattern of activities was identical to that observed in Figure 4a. To study the activities of the mutated promoters under HIF-1α stabilized conditions, the reporter constructs were transfected into MCF 7 cells that were then treated with CoCl_2_. As shown in Figure 4d reporter activities were enhanced following CoCl_2_ treatment without changing the pattern seen in Figure 4a. To further explore HIF-1 regulation of *DDX3* expression we co-transiently transfected MCF 7 cells with the mutated promoter-reporter constructs and a constitutive expressing HIF-1α vector construct (Figure 4e). Under these conditions all reporter activities roughly tripled and the loss of the HRE-1 site showed qualitatively the same diminishing effect on reporter activities. In total, the overall patterns of activities seen in Figure 4 remained qualitatively very similar regardless of the conditions tested and all these results indicate that loss of HRE-1 alone was sufficient to decrease reporter activity to roughly the same degree as what occurred when HRE-1 + 2 or +3 or HRE-1+2+3 were mutated. On the other hand, loss of either HRE-2 or HRE-3 or both HRE-2 + 3 either caused an increase in reporter activities or had no impact on reporter activity respectively.

### Chromatin immunoprecipitation assay for HIF-1α binding to the DDX3 promoter

**Figure 5:**
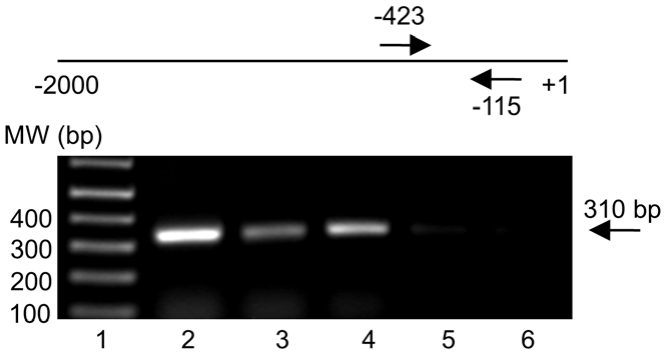
ChIP assay: *in vivo* binding of HIF-1 to the DDX3 promoter in MCF 10A cells. At the top of the gel is a schematic representation of the DDX3 promoter. Arrows flank the region (-423 to -115) amplified by PCR with DDX3 promoter specific primers. Gel shows: lane 1- molecular weight (MW) marker, lane 2- total input chromatin, lane 3-acetyl histone H3 precipitation, lane 4-anti-HIF-1α precipitation under hypoxic conditions, lane 5-anti-HIF-1α under normoxic conditions, and lane 6-anti-actin precipitation. Identical volumes from each final precipitation were used for PCR (except for the input chromatin, which was diluted 100x).

To further demonstrate that HIF-1 physically binds to the *DDX3* promoter and regulates *DDX3* gene activity, MCF 10A cells were exposed to hypoxia for 8 h and the binding of HIF-1 to *DDX3* promoter was analyzed using a ChIP assay. PCR was designed to amplify the region from −423 to −115 bp of the *DDX3* promoter, as schematically shown in Figure 5 DNA/protein complexes precipitated with an anti-HIF-1α antibody resulted in a detectable PCR-amplified DDX3 promoter-specific product only in the case of cells treated with hypoxia (Figure 5: lane 4). The positive controls: unprocessed total chromatin as well as anti-acetyl-histone precipitations (Figure 5: lane 2 and 3 respectively), also gave amplified products. However, no specific DDX3 amplified products were observed in preparations of DNA that were obtained from cells left in normoxia and the use of a nonspecific antibody during the precipitation step also gave a negative result (Figure 5: lane 5 and 6 respectively). Together, these results indicate that HIF-1 binds directly to the endogenous *DDX3* promoter in live cells under hypoxic conditions. Overall this experiment provides strong support of the promoter-reporter activity assays and indicates that at least one functional HRE sequence can be assigned to this region of the *DDX3* promoter.

### Gross co-localization of DDX3 and HIF-1α protein expression in xenograft tumor samples

**Figure 6:**
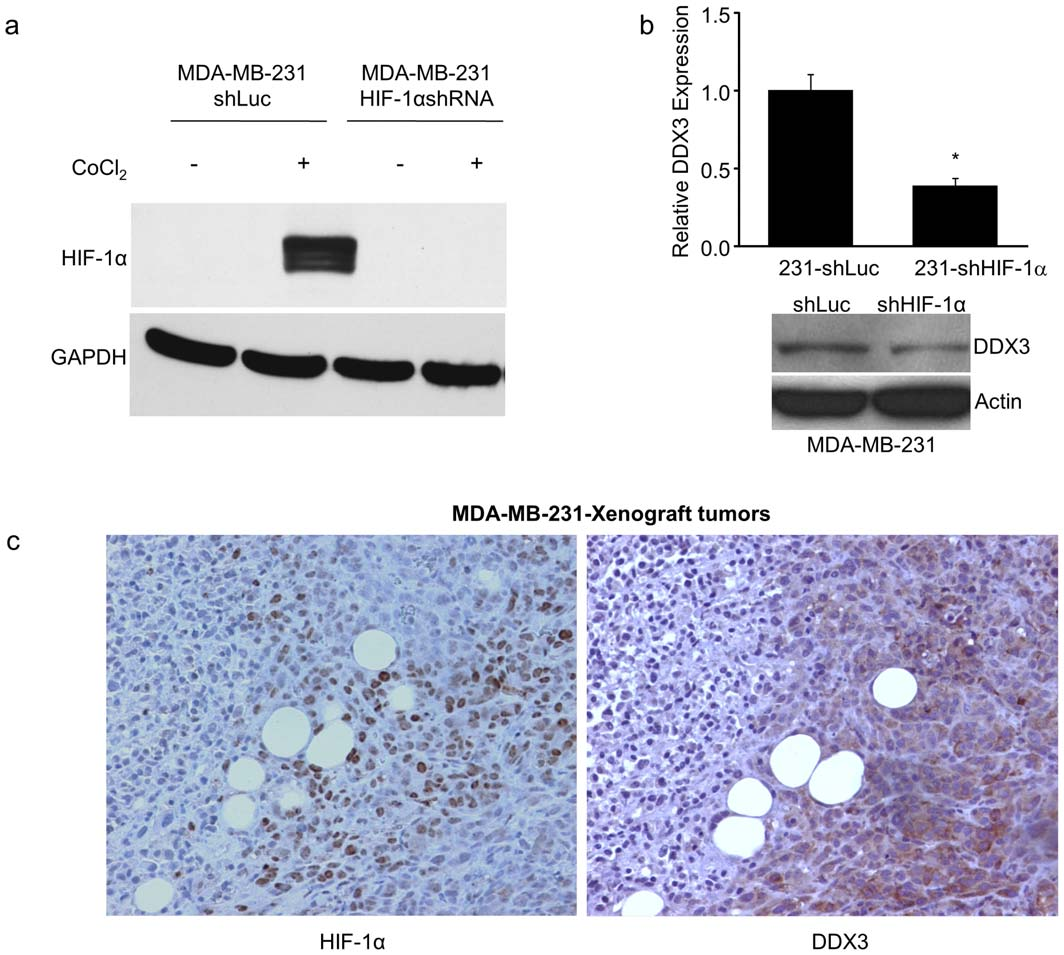
Correlation between HIF-1α and DDX3 protein expression in MDA-MB-231 breast cancer cells. (a) Immunoblot showing expression levels of HIF-1α following CoCl_2_ treatment in HIF-1α knockdown cells. (b) Histogram showing relative fold expression of DDX3 mRNA protein levels of MDA-MB-231 versus MDA-MB-231-shHIF-1α cells. Expression of DDX3 was downregulated in MDA-MB-231-shHIF-1α relative to MDA-MB-231 cells at both the mRNA and protein levels. (c) Immunohistochemical staining for HIF-1α and DDX3 in sequential slices of a MDA-MB-231 tumor xenograft. Tumor sections were stained with antibodies that are specific for HIF-1α and DDX3. Samples were counterstained with hematoxylin to reveal morphology. Error bars represent ±SD.

DDX3 and HIF-1α are highly expressed in the very aggressive breast cancer cell line, MDA-MB-231. To understand the possible HIF-1 mediated regulation of DDX3 in aggressive cancers, we knocked down HIF-1α levels in MDA-MB-231 using the lentiviral based shRNA described above. As shown in Figure 6a, HIF-1α was undetectable in MDA-MB-231-shHIF-1α under normoxia as well as during CoCl2 treatment, while being readily detectable in MDA-MB-231-shLuc cell lysates. To correlate the expression levels of DDX3 to that of HIF-1α levels, qRT-PCR and immunoblot scoring for DDX3 transcript and protein were performed on identical samples. Figure 6b shows that levels of DDX3 transcript and protein were reduced in MDA-MD-231-shHIF-1α cells. These results support the reporter assays and indicate that HIF-1 is a direct or indirect transcriptional regulator of DDX3 in aggressive breast cancer cells. We next examined if there is a correlation between DDX3 and HIF-1α protein expression in a mouse MDA-MB-231 xenograft model using anti-DDX3 and anti-HIF-1α antibodies to score for these proteins in immunohistochemical assays. Figure 6c shows a representative immunohistochemical staining of DDX3 and HIF-1α in sequential MDA-MB-231 tumor slices and the overlapping pattern of expression of these proteins within a growing tumor. Thus, much of the brown nuclear staining pattern of HIF-1α (right tumor slice), which is visible throughout much of the central and into the lower right regions of the slice, co-registers with the brown cytoplasmic staining pattern of DDX3 (left tumor slice) in an adjacent tumor slice and that the same areas of the slices (e.g., upper left regions) show no staining. This provides good evidence that HIF-1 may be contributing to the up-regulation of DDX3 expression in hypoxic regions of breast tumors.

## Discussion

Hypoxia is one of the major stress inducing factors that arises within solid tumors including breast tumors [40], [41]. A low oxygen condition stabilizes HIF-1α and HIF-2α and these can then dimerize with ARNT (HIF-1β) forming HIF-1 and HIF-2, which subsequently modulate gene expression programs that effectively promote cell survival [42], [43]. The importance of chronic hypoxia on cancer cells is that this condition has been associated with the generation of aggressive phenotypes, chemo- and radiation resistance, and metastatic potential [44]–[46]. Our recent studies have provided evidence that in breast cancer up-regulation of DDX3 expression can be associated with an aggressive phenotype and down-regulation or targeted inhibition of DDX3 can mitigate its actions [19]. Whether DDX3 is a contributing factor to the aggressive phenotype of hypoxic cancer cells is not known and the question of transcriptional regulation of human DDX3 during hypoxia has not previously been addressed. Given this, it was pertinent to ask about the molecular mechanisms that might induce DDX3 expression in response to hypoxia. In the present study, we provide the first evidence that hypoxia induces the expression of DDX3 in human breast epithelial cells and moreover that this regulation is mediated in part by HIF-1 binding to HRE(s) within the DDX3 promoter.

The *in vitro* increase in the expression of DDX3 mRNA measured in both MCF 10A and MCF 7 cell lines following hypoxic treatment occurred in a time dependent manner and was transient. This is consistent with other reports that have shown that measured increases in mRNA expression during hypoxia can be dependent on the severity as well as duration of the treatment and the stability of an mRNA in a given cell line [45], [47]. For example, Guo *et al.* reported that the induction of the *CYGB* gene in response to hypoxia was instantaneous but the time that the maximum mRNA level was reached varied with the cell line and thus was found at 3 h in BEAS-2B cells and 6 h in HeLa cells [48]. Maximum mRNA expression of DDX3 is observed in both cell lines tested here at 8 h of hypoxic treatment but in MCF 10A cells these levels declined at later time points, which was also the case during CoCl_2_ treatment of MCF 7 cells. Our immunoblot data mirrors the mRNA data in that DDX3 protein levels increased in both cell lines following 8 h of either hypoxia or CoCl_2_ treatment but reflect that the protein stability was higher than the mRNA stability in MCF 10A cells. Recent evidence has shown that HIF-1 and not HIF-2 is the principal HIF involved with the regulation of the genes in response to hypoxia in cancer cell lines including hepatoma, neuroblastoma, and breast cancer [49], [50]. Thus, we used shRNA knockdown of HIF-1α to determine the specificity of hypoxia induced DDX3 expression. The data obtained with the HIF-1α knockdown in MCF 10A and MCF 7 cell lines provide consistent evidence supporting the major involvement of HIF-1 and not HIF-2 in the transcriptional activation of *DDX3* by hypoxia and indicated that DDX3 could be considered a new member of the growing family of HIF-1 targeted genes.

In order to define the binding sites of HIF-1 in the DDX3 promoter that are contributing to increased expression under hypoxic conditions, we performed luciferase based reporter assays using 2 kb gene sequence that is immediately up stream of the translation start site [51]. In our luciferase reporter studies with MCF 7 cells we observed that the promoter fragment that extended approximately 1.5 kb upstream from the translation start site showed basal activity that was relatively high in comparison to the other promoter-reporter constructs tested. This is consistent with a similar high basal reporter activity reported during a characterization of the DDX3 promoter in two human embryonic carcinoma cell lines NEC8 and NEC14 [51]. This is likely due to the binding of basal levels of transcriptional activators to the upstream region of DDX3 promoter. When we co-transfected a constitutively expressing HIF-1α vector along with the promoter-reporter constructs the reporter activity increased by an order of magnitude for all the promoter constructs expect the two that lack the −320 bp region, i.e., the region closest to the translation start site. This indicates that HIF-1 directly or indirectly can regulate DDX3 expression via a *cis*-element(s) located within the −320 bp proximal promoter region that is particularly critical to the modulation of expression by HIF-1. An analysis of the 1.5 kb DDX3 promoter sequence showed that it contains at least 3 core hypoxia response elements (HREs), A/GCGTG, i.e., HIF-1 binding sites. Of the various mutated promoter-reporter constructs tested the mutation of the site we designated as HRE-1, located within the −320 bp region, appeared to have the largest impact on a decrease of reporter activity, under both normoxic and hypoxic mimic conditions. Recently, constitutive expression of HIF-1α was detected in MCF 7 cells under normoxia [52], which we also observed (see Figure 1d: lane 1). To understand if the high basal reporter activity is due to constitutive stabilization of HIF-1α in MCF 7 cells we estimated the reporter activity in MCF 7-shHIF1-α cell line. The reporter activities were decreased by ∼1.5 to 2 fold but were not completely ablated, which is likely due to promoter regulation by other transcription factors that are active on these portions of the DDX3 promoter. Despite the differences in the induction of luciferase activity qualitatively similar data was obtained during CoCl_2_ treatment as compared to untreated cells.

**Figure 7:**
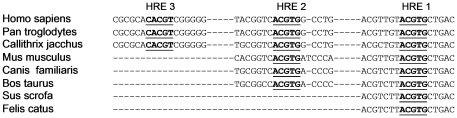
Comparison of regions of the DDX3 promoter among eutherian mammals. Conserved core hypoxia response elements (HRE-1, 2, & 3), which are putative HIF-1 binding sites (indicated in bold and underlined) are shown. HRE-1 and surrounding sequences are highly conserved across species.

To support the sequence-specific binding by HIF-1 to HRE-1 during hypoxic conditions within the nucleus of live cells we preformed ChIP assays. Overall, the mutated promoter-reporter and ChIP assays indicate that HIF-1 binds to an HRE in the proximal promoter of DDX3 under hypoxic conditions. Interestingly the proximal hypoxic responsive element close to the translation start site is highly conserved across eutherian mammals (Figure 7). This conservation of promoter elements is an indication that DDX3 expression in cells experiencing low O_2_ conditions is an important trait and that a functional DDX3 under these conditions is likely necessary during normal responses to hypoxia but may be pathologic during breast cancer progression. Indicative of this latter possibility is our observation that a portion of the DDX3 protein expression pattern overlaps with that of HIF-1α expression and that a similar lack of expression of both proteins is seen in other areas of the immunohistochemical staining of sequential tumor slices from a MDA-MB-231 xenograft model. In addition, the expression levels of DDX3 transcript and protein were reduced in HIF-1α knockdown MDA-MB-231 cell lines. These findings indicate that DDX3 may have important function(s) in the hypoxic environment of the tumor.

In summary, we have demonstrated, for the first time, that hypoxia is an inducer of DDX3 mRNA and protein expression in breast epithelial cell lines. Future studies aimed at the identification of the target genes under DDX3 regulation should provide important insights into a contributing molecular mechanism that modulates protein expression profiles during hypoxia. This will allow us to gain a better understanding of the protein repertoire that is modulated by hypoxia during breast tumorigenesis.

## Materials and Methods

### Cell lines and culture conditions

MCF 10A, MCF 7 and MDA-MB-231 cells (ATCC, Manassas, VA) were grown on standard tissue culture plastic ware in a 5% CO_2_-humidified incubator at 37°C. For hypoxia MCF 10A and MCF 7 cells were seeded at 1×10^6^ cells per 10 cm dish in duplicate. Twenty-four hours later the plates were transferred to modular incubators (Billups-Rothenberg Inc, USA) and flushed at 3 psi for 3 min with a mixture of 1% O_2,_ 94% N_2_, and 5% CO_2_. A modular chamber was used for each time point indicated in the figures. All chambers were transferred for culture to a standard 37°C incubator along with normoxic duplicate plates. In other experiments hypoxia mimetic conditions were chemically generated by treating cells with 200 µM cobalt chloride (CoCl_2_, Sigma, St. Louis, Missouri) for the indicated times.

### Quantitative real time PCR

Quantitative real time PCR was performed on RNA extracts from MCF 10A and MCF 7 cells. Total RNA was prepared from cultured cells under hypoxia (1% oxygen) for 0, 8, 12 and 24 h RNA was isolated according to the manufacturer's protocol (Qiagen) and reverse transcribed using a cDNA synthesis kit (Quanta BioSciences). The DDX3 mRNA sequence was amplified by real time PCR using DDX3 sense: 5′-GGAGGAAGTACAGCCAGCAAAG-3′ and antisense: 5′-CTGCCAATGCCATCGTAATCACTC-3′ primers. The relative expression of DDX3 in each cell line was normalized to that of hypoxanthine guanine phosphoribosyltransferase (HPRT) gene and gene expression in each sample was then compared with expression during normoxic growth conditions.

### RNA interference

To knock down HIF-1α expression in MCF 10A, MCF 7 and MDA-MB-231 cells, a lentiviral expression vector (pRRL) containing short hairpin (sh) RNA against HIF-1α mRNA was used. A lentiviral vector carrying a shRNA against luciferase (luc-shRNA) was used in the preparation of control virions.

### Reporter assays

Cells were plated 24 h before transfection at 1×10^5^ cells per well in a twenty four-well cell culture plate. Various DNA constructs (250 ng) and 5 ng of Renilla construct (Promega) were co-transfected using Trans IT-LT® (Mirus) transfection. For hypoxia mimetic assays, transfected cells were maintained in 200 µM CoCl_2_ for an additional 12 h. Following incubation, cells were lysed and luciferase activity was measured in a luminometer (Berthhold Detection System, Oak Ridge, TN). All experiments were performed in triplicate.

### Plasmid constructions and site-directed mutagenesis

Promoter deletions have been described previously [51]. Specific mutations were introduced into the luciferase reporter plasmids using QuikChange site-directed mutagenesis kits from Stratagene. Sequences of sense mutagenesis primers were as follows: HRE-1: 5′-GAGGGAGGGCACACGTTGTGGTACCTGACGTAGCCGGCTTTCC-3′, HRE-2: 5′-CGCACGCGACACCTACGGGGTACCGGCCTGCCGCCCTCTCAG-3′, and HRE-3: 5′-GCGGAGGCGCGCGCGCGATATCCGGGGGGTTGGCCAGG-3′ with mutated bases underlined. The generated plasmids were designated M1, M2, M3, M1+2, M1+3, M2+3 and M1+2+3 respectively. All base substitutions were verified by DNA sequencing.

### Chromatin immunoprecipitation assay

ChIP was carried out following established protocols. Chromatin complexes were immuno-precipitated using rabbit anti-HIF-1α or anti-histone deacetylase (positive control) antibodies. Samples prepared with anti-actin antibodies served as negative controls.

### Immunohistochemistry

Formalin-fixed, paraffin-embedded tumor tissues from MDA-MB-231 derived xenografts were used to study the expression pattern of DDX3 and HIF-1α. Sections were deparaffinized in xylene, blocked, and incubated with in-house generated polyclonal rabbit anti-DDX3 antibody (1∶5000) [16] and mouse or rabbit anti HIF-1α antibody (1∶1000, Novus Biologicals, CO, USA). Samples were then rinsed and incubated with horseradish peroxidase conjugated goat anti-rabbit whole IgG (Jackson ImmunoResearch Laboratories, West Grove, PA) and stained with DAB (Sigma). Sections were counterstained with hematoxylin Gill #3 (Sigma).
